# Surgical Strategy for the Chronic Achilles Tendon Rupture

**DOI:** 10.1155/2016/1416971

**Published:** 2016-10-25

**Authors:** Yangjing Lin, Liu Yang, Li Yin, Xiaojun Duan

**Affiliations:** Center for Joint Surgery, Southwest Hospital, Third Military Medical University, Chongqing 400038, China

## Abstract

*Background*. Chronic Achilles tendon rupture is usually misdiagnosed and treated improperly. This study aims to better understand the treatment of chronic Achilles tendon rupture.* Methods*. Patients who were not able to perform a single-limb heel rise were chosen. Pre- and postoperative magnetic resonance imaging (MRI) were conducted. By evaluating the presence or absence of Achilles tendon stumps and the gap length of rupture, V-Y advancement, gastrocnemius fascial turndown flap, or flexor halluces longus tendon transfer were selected for tendon repair. The function of ankle and foot was assessed by American Orthopaedic Foot & Ankle Society (AOFAS) ankle-hindfoot scores and Achilles Tendon Total Rupture Score (ATRS).* Results*. Twenty-nine patients were followed up. One patient had superficial incision infection, which was healed after debridement and oral antibiotics. Three months postoperatively, MRI showed some signs of inflammation, which disappeared at one or two years postoperatively. All patients were able to perform a single-limb heel rise. Mean AOFAS scores and ATRS scores were increased at the latest follow-up.* Conclusion*. Surgical options can be determined by evaluating the presence of the Achilles tendon stumps and the gap length, which can avoid using the nearby tendon and yield satisfactory functional results.

## 1. Introduction

The Achilles tendon is one of the most commonly ruptured tendons of the lower extremity [1–4]. Clinically, acute Achilles tendon rupture can be easily diagnosed and cured; however, a significant number of cases are still neglected without treatments. Chronic Achilles tendon rupture is usually defined as the rupture that occurs in 4 to 6 weeks after injury [3]. The symptoms of chronic Achilles tendon rupture include pain, decreased strength, fatigue, and ankle stiffness. During physical examination, a palpable gap between the rupture ends can be observed. Chronic Achilles tendon rupture often occurs 2 to 6 cm proximal to the stumps, but it sometimes can also be observed at the stumps [5]. Usually, small gaps (less than or equal to 2 mm) of chronic Achilles tendon rupture can be directly closed in an end-to-end manner [6]. However, there is still no standard treatment for chronic Achilles tendon rupture with large gaps [7]. Recently, Den Hartog [6] used an flexor halluces longus tendon (FHLT) transfer for all defects over 2 cm. But Park and Sung [8] deemed that gaps greater than 4 cm in chronic Achilles tendon rupture that underwent various reconstruction methods depending on the state of the remaining could achieve good outcomes.

Magnetic resonance imaging (MRI) has been a reliable medical image tool for diagnosing Achilles tendon rupture and other joint diseases preoperatively [9], and it has a strong hint in the individualized rehabilitation treatment and judgment of residual pain [10]. However, there were rare researches reporting clinical follow-up of chronic Achilles tendon rupture by MRI.

Carrying on retrospective study on patients with chronic Achilles tendon rupture in our department and postoperative evaluation by MRI, we have provided the reference of the standardized treatment for future.

## 2. Materials and Methods

**Figure 1:**
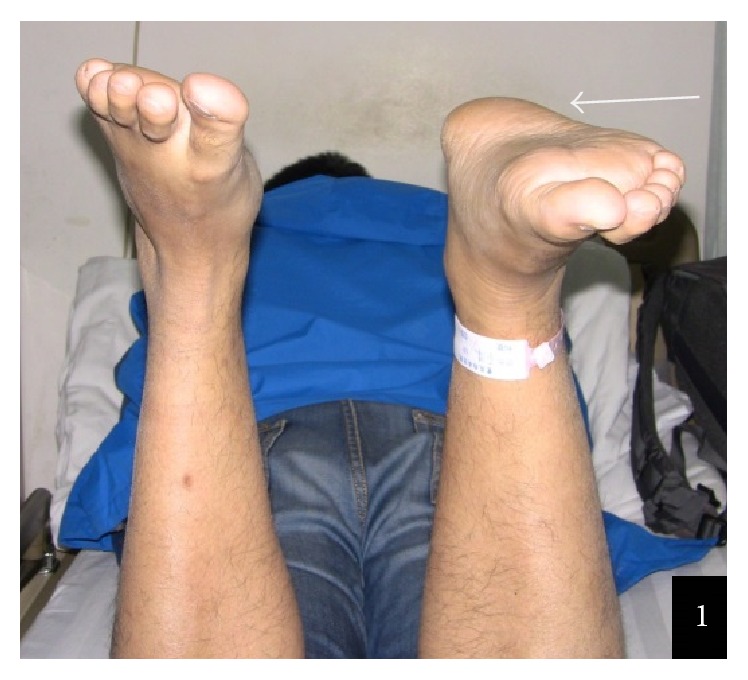
The right foot (arrow) was dropped because of Achilles tendon rupture when patient took prone position.

Retrospective analysis of the chronic Achilles tendon rupture cases in our department between January 2004 and July 2015 was conducted. Inclusive criteria were as follows: firstly, there is history of trauma at Achilles tendon; secondly, the interval from rupture to surgery was more than 4 weeks; thirdly, patients were not able to perform a single-limb heel rise; fourthly, MRI confirmed the final clinical diagnosis. Exclusion criteria were as follows: firstly, open Achilles tendon rupture; secondly, the history of local infection near the Achilles tendon rupture; thirdly, concomitant diseases with fracture, blood vessels rupture, or nerve rupture; fourthly, the patients who could not accept regular follow-up. The main preoperative physical signs included the following: firstly, there is localized tenderness; secondly, when the patient lied prone with the knee bent at 90°, the static position of ankle dorsiflexion was different between the normal and injury ankles (Figure 1); thirdly, with further squeezing the calf on both sides, passive plantar flexion should be present on the healthy side but absent on the injured side; fourthly, patients were not able to perform a single-limb heel rise with the injured lower extremity. X-ray test was the routine before operation and it could rule out the chance of fracture. Preoperative MRI was conducted with 0.2 T Artoscan C (Italy) by using dedicated coil, and all MRIs showed that the Achilles tendon was not in continuity in transaxial and sagittal planes.

In 12 cases, acute Achilles tendon rupture had been neglected after the first injury. The other 17 cases with correct first-time diagnosis had Achilles tendon rupture after failure of conservative treatment. Of all the involved patients, 23 cases were male and 6 cases were female, with the mean age of 40.3 years (range 19.2–71.5 years) at surgery. 16 cases had left Achilles tendon rupture and the other 13 cases had right Achilles tendon rupture (Table 1).

### 2.1. Surgical Strategy

**Figure 2:**
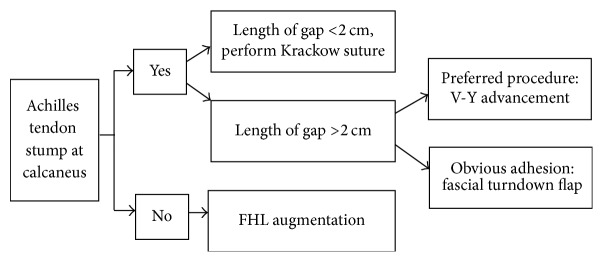
Treatment strategy for chronic Achilles tendon rupture.

Appropriate method for tendon reconstruction was chosen based on preoperative MRI results (e.g., presence or absence of Achilles tendon stumps) as well as the defect gap measured during operation. If the tendon stumps had enough integrity of the Achilles tendon, ruptures of the gap less than 2 cm could be repaired directly by Krakow method, while the gap greater than 2 cm could be addressed through V-Y advancement or gastrocnemius fascial turndown flap. If the tendon stumps did not have enough integrity of the Achilles tendon, FHLT transfer could be considered for reconstruction (Figure 2).

### 2.2. Surgical Technique

All operations were performed by two senior orthopedic surgeons. The patient was placed prone on the operating table. Anesthesia of lumbar plexus-sciatic nerve block and thigh tourniquet were used. A posterolateral or posteromedial incision was made over the position of the Achilles tendon rupture. The Achilles tendon was adequately exposed. The surrounding tissue and distal tendon stumps were carefully preserved. For the V-Y advancement [11, 12], V-shape part was designed in aponeurosis; limbs of the V-shape part should be attached to soleus as much as possible; the tendon should be slowly torn with caution. For the gastrocnemius fascial turndown flap [13, 14], the gap of ruptures should be measured; the length of the turndown flap was then determined as 2 cm in addition to the length of gap. FHLT could be considered for reconstruction. The main operation technique included harvesting the FHLT and transferring it into the bone tunnels drilled in posterior calcaneus. Hydroxyapatite composite screws (Smith & Nephew) were used to fix the FHLT to the bone tunnel [15, 16]. The ankle was kept in appropriate position. After reconstruction of Achilles tendon rupture, the wound was closed in layers.

### 2.3. Postoperative Treatment

The ankle was kept in plantar flexion (up to 20°) for 4–6 weeks using a below-knee cast; then the cast angle in plantar flexion was decreased in nonpain conditions. Patients were encouraged to perform physical exercises under rehabilitation guidelines. 6 weeks after operation, partial-weight-bearing crutch ambulation was allowed with ankle-foot boot. At 12 weeks postoperatively, patients were allowed to participate in riding and swimming without restrictions. Patients can do strenuous sports such as running and jumping at 1 year postoperatively.

### 2.4. Statistical Analysis

All patients were asked to accept regular follow-up, during which the MRI and physical examination were applied. The Achilles tendon function was evaluated by integrity, pain, ankle strength, and range of motion. Postoperative complications such as wound healing problems were also observed. At the latest follow-up, subjective outcomes including the American Orthopaedic Foot & Ankle Society (AOFAS) ankle-hindfoot scores [8] and Achilles Tendon Total Rupture Score (ATRS) [14] were reevaluated. Comparisons between the preoperative and postoperative AOFAS and ATRS scores were performed with use of paired-sample *t*-tests in a commercial statistics package (version 18.0; IBM, Chicago, IL, USA). Significance level was set as *P* < 0.05.

## 3. Results

**Figure 3:**
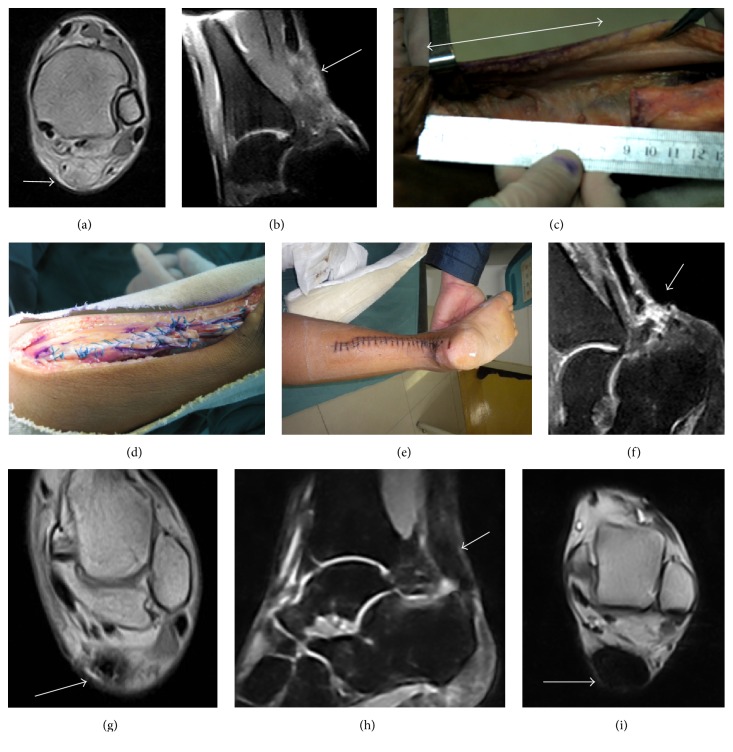
A 23-year-old male. The interval from rupture to surgery was 7 weeks. Preoperative MRI showed chronic Achilles tendon rupture (arrow) (a, b). Length of gap (double-headed arrow) was 9 cm after scar tissue debridement (c). V-Y advancement performed (d). Wound healed (e). MRI showing inflammation (arrow) at 6 weeks of follow-up (f, g). MRI showing fusiform-shaped tendon thickening and homogeneous low-signal at 1 year postoperatively (arrow) (h, i).

**Figure 4:**
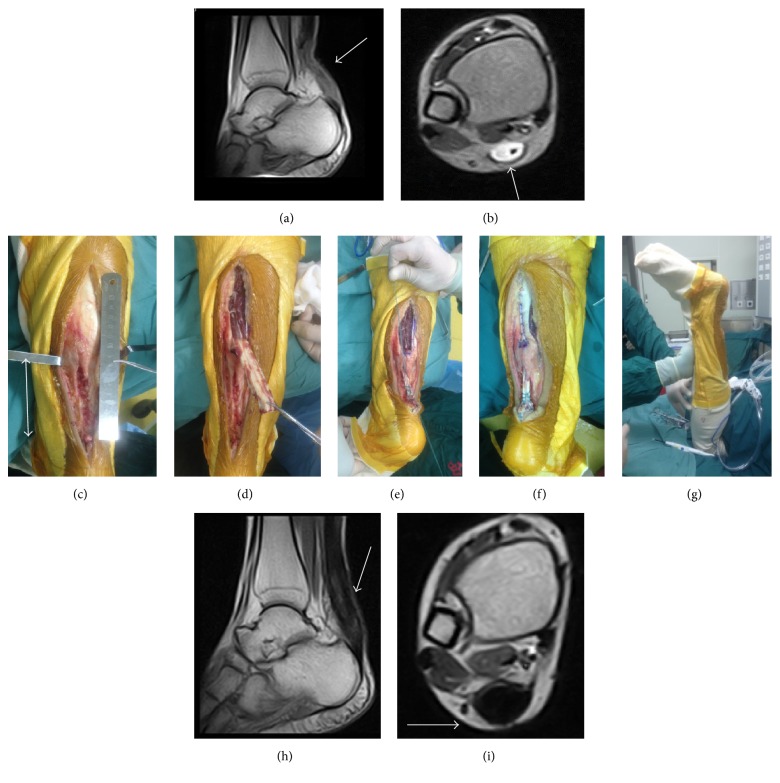
A 30-year-old male. The interval from rupture to surgery was 22 weeks. Preoperative MRI showed chronic Achilles tendon rupture (arrow) (a, b). Length of gap (double-headed arrow) was 9 cm, a lot of scar tissue located stump area, and it was difficult to perform V-Y advancement (c). Gastrocnemius fascial turndown flap performed (d, e). Sutured with the stump and adjusted the tension (f). The position of ankle was similar to the other side after surgery (g). MRI showing fusiform-shaped tendon thickening and homogeneous low-signal at 1 year postoperatively (arrow) (h, i).

**Figure 5:**
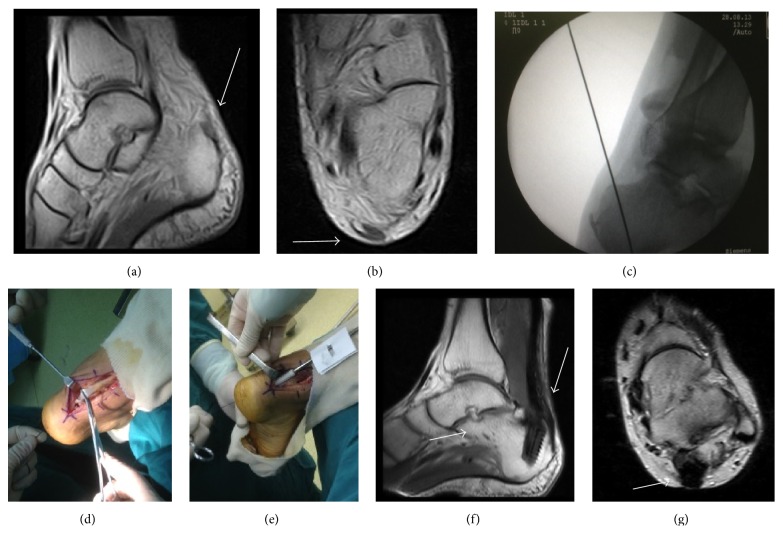
Preoperative MRI showed chronic Achilles tendon rupture and no stump at calcaneus (a, b). FHLT transfer performed (c, d). Using the screws of hydroxyapatite composition to fix FHL into the bone tunnels (e). MRI showing the continuity of the Achilles tendon and homogeneous low-signal at 34 months postoperatively (arrow) (f, g).

No rupture gap was less than 2 cm. Gaps greater than 2 cm (18 cases) were addressed through V-Y advancement, including 7 cases whose rupture gaps were more than 6 cm (Figure 3). 8 cases received gastrocnemius fascial turndown flap, including 4 cases whose rupture gaps were more than 6 cm (Figure 4). 3 cases had undergone FHLT transfer (Figure 5). The mean follow-up period was 31 months (range 13–68 months).

All patients were followed up. One patient had superficial incision infection, which was healed after debridement, dressing change, and oral antibiotics. All patients were able to perform a single-limb heel rise and had returned to their preinjury level of sports participation. Three months after operation, MRI showed some signs of inflammation, which disappeared at one and two years postoperatively. At the latest follow-up, MRI showed continuity of the Achilles tendon. Mean AOFAS scores increased from 60.13 ± 10.76 points preoperatively to 94.63 ± 4.02 points at the latest follow-up (*P* < 0.05). Mean ATRS score also showed significant improvements from 43.83 ± 11.78 points preoperatively to 92.62 ± 7.77 points at the latest follow-up (*P* < 0.05).

## 4. Discussion

The treatment of chronic Achilles tendon rupture is a challenge for most orthopedic surgeons [17–19]. It is different from the acute Achilles tendon rupture in pathophysiology. Chronic Achilles tendon ruptures with large gaps may lead to ankle dysfunction [1–3, 20, 21]. If the gap of rupture is bridged by scar tissue, ankle weakness and gait disturbances may occur due to severely infiltrated fat composition [7]. Though chronic Achilles tendon rupture is not unusual, it is frequently misdiagnosed. According to previous study, neglected Achilles tendon ruptured can occur at rate as high as 20% clinically [22]. After Achilles tendon rupture occurred, the strength of plantar flexion is reduced [23], and the patients are not able to perform a single-limb heel rise with the injured lower extremity. The sign is the vital indication for reconstruction surgery. In the current study, the modified Thompson test was used to diagnose the Achilles tendon rupture. When the patient lied prone with the knee bent at 90°, Thompson test could get higher positive rate than the knee being straight. Surgical reconstruction could restore full strength of the Achilles tendon and thus improve the activity level of patients [7]. However, the reconstruction and augmentation of chronic Achilles tendon rupture are complex, and they might affect the choice of procedures. In the literature, no optimal treatments for chronic Achilles rupture had been documented. Our study provided a simple and useful treatment strategy for chronic Achilles tendon rupture. Based on the presence or absence of Achilles tendon stumps, the appropriate reconstruction methods can be determined. Our results in the study supported the surgery integrity.

The optimal technique for treating chronic Achilles tendon rupture was controversial [24–27]. V-Y advancement flap was first introduced by Abraham and Pankovich, as a treatment for neglected Achilles tendon rupture [11]. In our study, V-Y advancement was used in 18 cases, in which the maximal gap was 9 cm (range from 3 to 9 cm). In these patients, the AOFAS and ATRS scores showed significant improvements at latest follow-up and no serious complications were observed. Ahmad et al. [7] deemed that gaps greater than 6 cm in chronic Achilles tendon rupture could be a big challenge to surgeons. In their study, defect up to 6 cm could be repaired; 75% of the patients had regained full tendon strength and could perform heel raises [11]. Khiami et al. [25] suggested that V-Y advancement flap was appropriate for gaps of 3 to 5 cm when the reconstruction was delicate. McClelland and Maffulli [18] reported that V-Y advancement could achieve satisfactory results in treating chronic Achilles tendon ruptures measured over 6 cm in length. Our results also supported these conclusions.

Gastrocnemius fascial turndown flap is also a useful technique in repairing chronic Achilles tendon ruptures with great defect. In 1931, Christensen [23] first reported his method in which the defect was filled using a fascial turndown flap sized 2 cm by 10 cm. In our study, the maximal gap treated with this technique was 10 cm, and the patient had achieved satisfactory ankle function. Other authors have also described successful repair of chronic Achilles tendon ruptures with modified gastrocnemius fascial turndown flaps. Tay et al. [13] reported that chronic Achilles tendon rupture was treated with two turndown flaps and FHL augmentation yielded satisfactory results during two-year follow-up. Peterson's et al. study [5] also revealed similar outcomes when treating central defect of approximately 12 cm.

Three aspects should be considered in V-Y advancement reconstruction for chronic Achilles tendon rupture. First, V-Y advancement is more suitable for acute chronic Achilles tendon rupture; second, V-Y advancement should be used in young patients; third, soleus muscle can provide revascularization for the tendon of V-Y advancement so that the Achilles tendon rupture can heal faster. In comparison, gastrocnemius fascial turndown flap was suitable for all kinds of patients, but the rupture may heal more slowly because soleus muscle cannot directly provide revascularization. In our opinion, V-Y advancement should be chosen with priority for the ruptures with a gap greater than 2 cm. When V-shape part was not enough in the operation and the soleus cannot attach to V-shape part, fascial turndown flap was a choice for reducing the operative wound.

FHLT transfer was first used to treat chronic Achilles tendon rupture about 20 years ago [16]. It had developed to a widespread application in tendon stumps reconstruction. When the tendon stumps did not have enough integrity of the Achilles tendon, we can consider FHLT for surgical reconstruction. The main operation technique included harvesting the FHLT and transferring the FHL into the bone tunnels made in posterior calcaneus. Therefore, the FHL muscle can generate enough strength to raise the heel and substitute the function of Achilles tendon. FHLT has four advantages in the chronic Achilles tendon reconstruction. First, it is close to the Achilles tendon; second, the FHL muscle has the same function as the triceps surae; third, it has adequate strength; fourth, it has the same axis of moving with the Achilles tendon [16]. Since FHLT has the above advantages in the chronic Achilles tendon reconstruction, more and more surgeons preferred this surgical technique and had reported positive outcomes. Yeoman et al. [15] treated 11 patients with chronic Achilles tendon rupture using FHL technique and interference screw fixation, showing reliable outcomes and low complication morbidity. Oksanen et al. [28] reported that mean hypertrophy of 52% of the FHL muscle was observed by MRI after FHL. Similar phenomenon was also observed in the current study. This may demonstrate that the FHL had good adaptation capacity [28]. Coull et al. [29] found that though the active range of motion of interphalangeal joint might bet lost, it did not impair functions such as walking, running, and stair climbing. But, sometimes, this technique can reduce the function of halluces; therefore, it should not be routinely recommended for young patients. Besides, Achilles tendon stump is very important for Achilles tendon reconstruction because it has the normal tendon bone interface structure and Sharpey's fiber [30], whereas, in FHLT transfer, there would be a rather long process of interface structure reconstruction [31]. Thus, we suggested that FHLT transfer only be used for increasing strength when there were not enough tendon stumps.

We also found that MRI could be a useful tool for diagnosing Achilles tendon rupture. It can assess the integrity of the Achilles tendon stumps, acquire the information of Achilles tendon healing, and so on. No patient was misdiagnosed by MRI in our study. Postoperative follow-up MRI had a strong hint in the individualized rehabilitation treatment and judgment of residual pain. In our study, three months after operation, MRI showed some signs of inflammation, which disappeared at one and two years postoperatively.

One limitation of the current study was that it was a retrospective study with small number of cases. Also, we did not have isokinetic strength analysis of the patients, nor did we conduct comparative study among the three techniques. Future study with larger number of cases and longer follow-up time would be able to provide stronger evidence in a multicenter randomized control clinical trial.

## 5. Conclusion

A clear treatment strategy can be determined by evaluating the presence or absence of the Achilles tendon stumps and the gap length of rupture after Achilles tendon rupture, which can avoid using the nearby tendon and yield satisfactory functional results by making the most of the local Achilles tendon and gastrocnemius fascial. MRI is used in the healing process observation and is a useful tool for postoperative rehabilitation.

## Figures and Tables

**Table 1:** Characteristics of patients.

Characteristics	Patients (*n* = 29)
*Sex*	
Male	23
Female	6
*Age (yr)*	40.3 (19.2 to 71.5)^*∗∗*^
*Side*	
Right	13 (44.8%)^*∗*^
Left	16 (55.2%)^*∗*^
*Reasons for chronic rupture *	
Neglected	12 (41.4%)^*∗*^
Failed treatment	17 (58.6%)^*∗*^
*Time from injury to surgery (wk)*	13 (4 to 104)^*∗∗*^
*Length of gap (mm)*	56 (25 to 100)^*∗∗*^
*Follow-up (mo)*	31 (13 to 68)^*∗∗*^

^*∗*^The values are given as the number of patients, with the percentage in parentheses.   ^*∗∗*^The values are given as the mean, with the range in parentheses.
